# Genome-Wide Analysis of Calmodulin Binding Transcription Activator (CAMTA) Gene Family in Peach (*Prunus persica* L. Batsch) and Ectopic Expression of *PpCAMTA1* in Arabidopsis *camta2,3* Mutant Restore Plant Development

**DOI:** 10.3390/ijms231810500

**Published:** 2022-09-10

**Authors:** Can Yang, Zhihao Li, Xiangmei Cao, Wenyi Duan, Chunyan Wei, Chi Zhang, Dan Jiang, Mengtao Li, Kunsong Chen, Yongjin Qiao, Hongru Liu, Bo Zhang

**Affiliations:** 1 Laboratory of Fruit Quality Biology/Zhejiang Provincial Key Laboratory of Horticultural Plant Integrative Biology, Zhejiang University, Hangzhou 310058, China; 2 Institute of Crop Breeding & Cultivation Research, Shanghai Academy of Agricultural Sciences, Shanghai 201403, China; 3 Shandong (Linyi) Institute of Modern Agriculture, Zhejiang University, Linyi 276000, China

**Keywords:** CAMTA, peach fruit, cold, salicylic acid, UV-B, plant immunity

## Abstract

Calmodulin-binding transcription activator (CAMTA) is a transcription factor family containing calmodulin (CaM) binding sites and is involved in plant development. Although CAMTAs in Arabidopsis have been extensively investigated, the functions of CAMTAs remain largely unclear in peaches. In this study, we identified five peach CAMTAs which contained conserved CG-1 box, ANK repeats, CaM binding domain (CaMBD) and IQ motifs. Overexpression in tobacco showed that PpCAMTA1/2/3 were located in the nucleus, while PpCAMTA4 and PpCAMTA5 were located in the plasma membrane. Increased expression levels were observed for *PpCAMTA1* and *PpCAMTA3* during peach fruit ripening. Expression of *PpCAMTA1* was induced by cold treatment and was inhibited by ultraviolet B irradiation (UV-B). Driven by *AtCAMTA3* promoter, *PpCAMTA1/2/3* were overexpressed in Arabidopsis mutant. Here, we characterized peach *PpCAMTA1,* representing an ortholog of *AtCAMTA3.* *PpCAMTA1* expression in Arabidopsis complements the developmental deficiencies of the *camta2,3* mutant, and restored the plant size to the wild type level. Moreover, overexpressing *PpCAMTA1* in *camta2,3* mutant inhibited salicylic acid (SA) biosynthesis and expression of SA-related genes, resulting in a susceptibility phenotype to *Pst* DC3000. Taken together, our results provide new insights for *CAMTAs* in peach fruit and indicate that *PpCAMTA1* is associated with response to stresses during development.

## 1. Introduction

Calcium ion (Ca^2+^), as a best characterized second messenger, is involved in diverse physiological processes in plants [1]. For fruit, the complex relationship between calcium, water, cell walls and signaling pathways make calcium an essential player in fruit physiology and development [2]. Calmodulin (CaM) is the important Ca^2+^ receptor [3], the expression of CaMs in papaya fruit showed responses to temperature stresses and during fruit development [4]. CaM-binding transcription activator (CAMTA), also named as signal responsive (SR), receives the unambiguous alerts of calcium influxes by CaMs, which is considered to be the largest and best characterized CaM-binding TF family for Ca^2+^ signaling in plants [5]. Tobacco ethylene response gene (*NtER1*) is the earliest *CAMTA* gene identified in plants [6]. Later, at least 465 *CAMTA* genes were identified from 112 plants [7]. Plant CAMTA proteins have several conserved functional domains, including a DNA-binding domain (CG-1), a transcription-associated immunoglobulin domain (TIG), ankyrin repeat domain (ANK), a calmodulin-binding domain (CaMBD) and several IQ motifs [8]. The CaMBD is a characteristic of plant CAMTAs, required for CaM binding and formed amphipathic α-helix structure [9,10]. Approximately one-quarter of plant CAMTA genes belonged to non-TIG type based on the absence of the TIG domain [7,11].

*Arabidopsis thaliana* has six CAMTA proteins [9], whose biological roles have been determined using loss-of-function mutants. AtCAMTA3 loss-of-function (*camta3*) mutants exhibit stunted growth and constitutive activation of immune responses [12,13]. These autoimmune phenotypes are more obvious in *camta2,3* double mutants and extreme in *camta1,2,3* triple mutants [14,15], suggesting AtCAMTA1, AtCAMTA2 and AtCAMTA3 are redundant negative regulators of plant immunity. In plants grown at warm temperature, AtCAMTA1, AtCAMTA2 and AtCAMTA3 repress the expression level of salicylic acid (SA) immunity pathway genes in an additive manner [14]. These genes include *ICS1* (*isochorismate synthase 1*, encoding the primary rate-limiting enzyme in SA biosynthesis in Arabidopsis) [16], and four genes encoding positive regulators of *ICS1*: *CBP60g* (*calmodulin binding protein 60-like.g*), *SARD1* (*SAR deficient 1*), *EDS1* (*enhanced disease susceptibility 1*) and *PAD4* (*phytoalexin deficient 4*) [17]. The transcript levels of these genes are much higher in *camta3*, *camta2,3* and *camta1,2,3* mutants, resulting in higher SA content and the induction of *PR1* (*pathogenesis related 1*, a marker gene for SA-mediated immune response) [12,14]. AtCAMTA1/2/3 also repress the biosynthesis of pipecolic acid (Pip) and *N*-hydroxypipecolic acid (NHP) in healthy plants, coordinated with SA pathway to optimize plant immune response [17,18]. In terms of abiotic stress response, CAMTA1 and CAMTA2 work in concert with CAMTA3 at low temperatures to induce the transcript levels of *CBF1*, *CBF2* and *CBF3* [14], CAMTA3 and CAMTA5 respond to a rapid decrease in temperature and increase the *DREB1s* expression [19]. The *camta1* mutant showed drought sensitivity [20] and hyper-responsive to auxin [21]. The *camta6* mutants displayed reduced sensitivity to salt and ABA during germination [22].

Apart from Arabidopsis, there are growing evidence that CAMTAs are key players in plant development and stimuli responses in other plants [5,23,24]. For example, virus-induced gene silencing (VIGS)-based knockdown of *SlSRs* in tomato showed that *SlSR1* and *SlSR3L* negatively regulate disease resistance response and *SlSR1L* positively modulates drought stress tolerance [25]. For horticultural crops, genome-wide analysis of *CAMTA* genes has been reported in several species, including tomato [10], grape [26], strawberry [27], citrus [28], banana [29] and durian [30]. At present, the functions of *CAMTAs* in fruit are mainly based on gene expression pattern analysis. For example, the distinct ripening-associated expression pattern of *CAMTA* genes in tomato and durian indicated their possible roles in fruit ripening [10,30]. Despite great progress being made in the analysis of *CAMTAs* gene family, our understanding of functions for fruit *CAMTAs* remains largely incomplete.

The peach (*Prunus persica* L. Batsch) is not only a popular economic crop in the world, but also a model plant for *Rosaceae* [31]. Peach fruit are highly susceptible to low temperature [32], pathogenic infection and physiological deterioration during ripening and storage [33]. *CAMTA* gene family may be a crossover point where calcium signaling intersects the ethylene, salicylic acid, immunity and cold signal transduction pathways, all of these signaling pathways have a major impact on fruit ripening and quality [2]. To date, studies investigating the *CAMTA* gene family in peaches are still lacking. Here, the hypothesis is that peaches contain multiple CAMTA genes and there should be one gene member represents an ortholog of Arabidopsis *AtCAMTA3* which is involved in developmental process.

In order to study the expression patterns and to characterize the potential functions of peach *CAMTAs*, bioinformatics methods were used to analyze the gene structure and chromosomal distribution, RNA-sequencing (RNA-seq) and quantitative reverse-transcription PCR (RT-qPCR) were applied to investigate stress-induced and spatiotemporal expression pattern of *CAMTAs*. We also demonstrate the plasma membrane localization of two peach *CAMTAs* instead of in the nucleus. Moreover, we carried out the ectopic expression of the peach *CAMTAs* under the control of the native *AtCAMTA3* promoter in *camta2,3* mutant. Our result showed that overexpression of *PpCAMTA1* complements the developmental defects of *camta2,3* mutant.

## 2. Results

### 2.1. Genome-Wide Identification and Protein Properties Analysis of CAMTAs in Peach

Five CAMTA members were identified in the peach genome database using BLASTP program with six Arabidopsis CAMTA protein sequences as queries. Same number of CAMTAs was also identified in various genome assemblies of different peach varieties (Table S1). In this study, the CDS sequences of these five genes were cloned and sequenced, then used for subsequent analysis. The information for the peach *CAMTAs* gene ID, protein length, molecular weight (MW), isoelectric point (pI) and grand average of hydropathicity (GRAVY values) were listed in Table 1. The peach CAMTA proteins varied in length from 914 to 1131 amino acids (aa), which resulted in variations in MW. The pI values of five CAMTAs range from 5.58 to 6.69, and the instability index (II) varies between 39.54 and 44.84. These proteins were considered as hydrophilic with the negative GRAVY values (Table 1).

Next, we extend the information of *CAMTA* genes to other *Rosaceae* species. The results showed that fruit crops from *Prunus* species (sweet cherry, almond and apricot) have similar CAMTAs number with peaches, which were 5, 5 and 4, respectively (Table 2 and Table S2). The number of CAMTA genes of strawberry from *Rosaceae* family is 16, which is more than three times than that of peach (Table 2 and Table S2). Here, we identified a total of 53 CAMTAs from *Rosaceae* family fruit crops, including strawberry, pear, apple, sweet berry, almond, apricot and peach.

### 2.2. Phylogenetic, Conserved Domain and Motifs Analysis of CAMTA Genes in Peach

**Figure 1:**
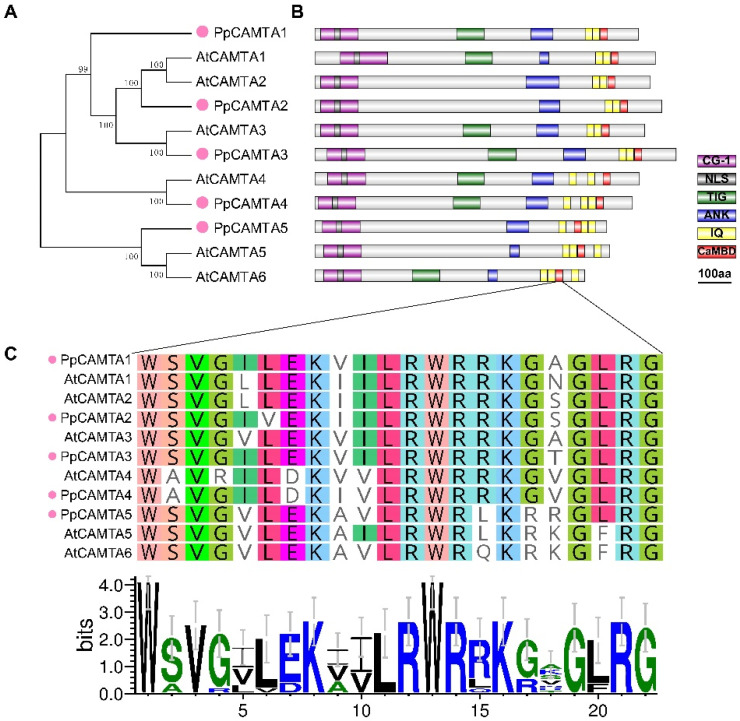
Phylogenetic relationship and conserved protein domains between peach and Arabidopsis CAMTAs. (**A**) Phylogenetic analysis of the peach and Arabidopsis CAMTAs. Peach CAMTAs were marked with pink dots. (**B**) Protein conserved domain composition in peach and Arabidopsis CAMTAs. Different protein domains were indicated in different colors. (**C**) Alignment of the CaMBD sequence between peach and Arabidopsis CAMTAs. The sequence logo of the functional residues in CaMBD domain of these CAMTAs was displayed below.

The phylogenetic tree was constructed to investigate the evolutionary relationship among Arabidopsis CAMTAs and peach CAMTAs (Figure 1A). Based on the phylogenetic tree, CAMTAs were divided into three groups. PpCAMTA1, PpCAMTA2 and PpCAMTA3 were distributed in a same clade with AtCAMTA1 to AtCAMTA3. PpCAMTA4 showed high sequence similarity with AtCAMTA4, while PpCAMTA5 was grouped with AtCAMTA5 and AtCAMTA6. Furthermore, the conserved domains of the peach CAMTA proteins were visualized (Figure 1B). Analysis of protein structures showed that they contained the essential conserved domains, including CG-1 box, ANK repeats domain, CaM binding domain (CaMBD) and IQ motifs. PpCAMTA2 and PpCAMTA5 were found to contain no TIG domain and were grouped into non-TIG type. The CaMBD amino acids of peach CAMTAs have high sequence similarity with their homologs in Arabidopsis (Figure 1C). The greatest similarity of the sequences was found between PpCAMTA4 and AtCAMTA4 with only two amino acids difference. The sequence logo for CaMBD in these CAMTAs was shown below the alignment (Figure 1C).

To further examine the structural features of peach CAMTAs, the conserved motifs were analyzed using the online server, MEME Suite. A total of 13 motifs were found in peach CAMTAs (Figure S1). Among them, 11 motifs existed in all peach CAMTAs, and some motifs corresponded to protein conserved domains. For example, motifs 1, 5 and 10 were linked to the conserved CG-1 domain, motif 2 and 13 partly overlapped with IQ domain and CaMBD domain. Conserved domains and motifs indicated the conservatism of these identified peach CAMTA family members.

### 2.3. Exon-Intron Structure Analysis and Chromosomal Locations of Peach CAMTAs

**Figure 2:**
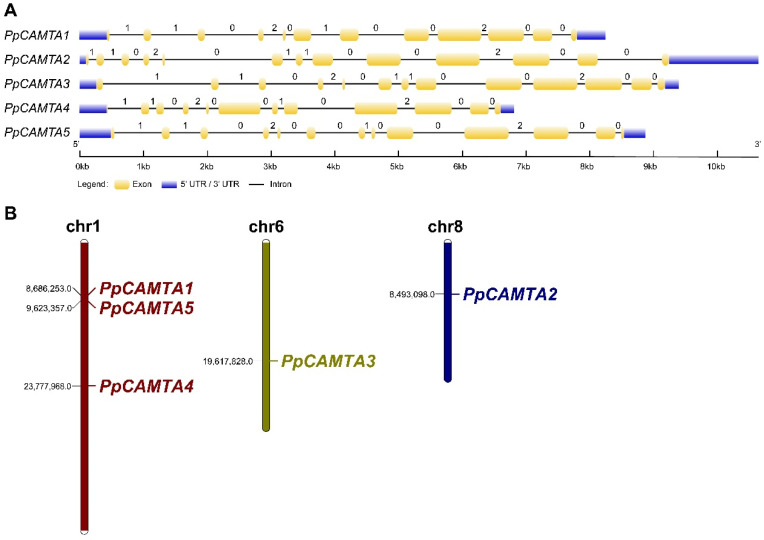
Distributions of exon-intron and chromosomal locations of peach *CAMTA* genes. (**A**) Distributions of exon-intron of peach CAMTA genes. Blue boxes represent UTR regions, black lines represent introns and yellow boxes represent exons. The numbers on introns indicate their phase type: Phase 0 intron does not disrupt a codon, phase 1 intron disrupts a codon between the first and second bases, and phase 2 intron disrupts a codon between the second and third bases. (**B**) Chromosome distributions of peach CAMTA genes. The chromosome number was shown at the top of each chromosome.

To get gene structure information of peach *CAMTA*s, the intron-exon structures were analyzed. As shown in Figure 2A, *PpCAMTA2*, *PpCAMTA3* and *PpCAMTA5* had 12 introns, while *PpCAMTA1* and *PpCAMTA4* contained 11 introns. Different numbers of introns were consistent with that of most plant species (10 to 13 introns) [7]. For the intron phases of five peach *CAMTA* genes, an intron phase pattern of ‘1102-0110-0200′ was observed in *PpCAMTA2* and *PpCAMTA3*, fitting with one of the most common distribution modes across plant *CAMTA* genes. However, *PpCAMTA1*, *PpCAMTA4* and *PpCAMTA5* showed different patterns with ‘1102-010-0200’, ‘1102-001-0200’ and ‘1102-0010-0200’, respectively. Similar to previous studies [7], our results showed that the lengths of the exons corresponding to intron phase pattern ‘0200’ were significantly longer than those corresponding to intron phase pattern ‘1102’. Chromosomal locations of peach *CAMTA* genes revealed that the genes were unevenly distributed on three chromosomes (Figure 2B). Most *CAMTA* members (*PpCAMTA1*, *4* and *5*) were located on chromosome 1, *PpCAMTA3* on chromosome 6, *PpCAMTA2* on chromosome 8.

### 2.4. Promoter Cis-Acting Regulatory Elements of Peach CAMTA Genes

**Figure 3:**
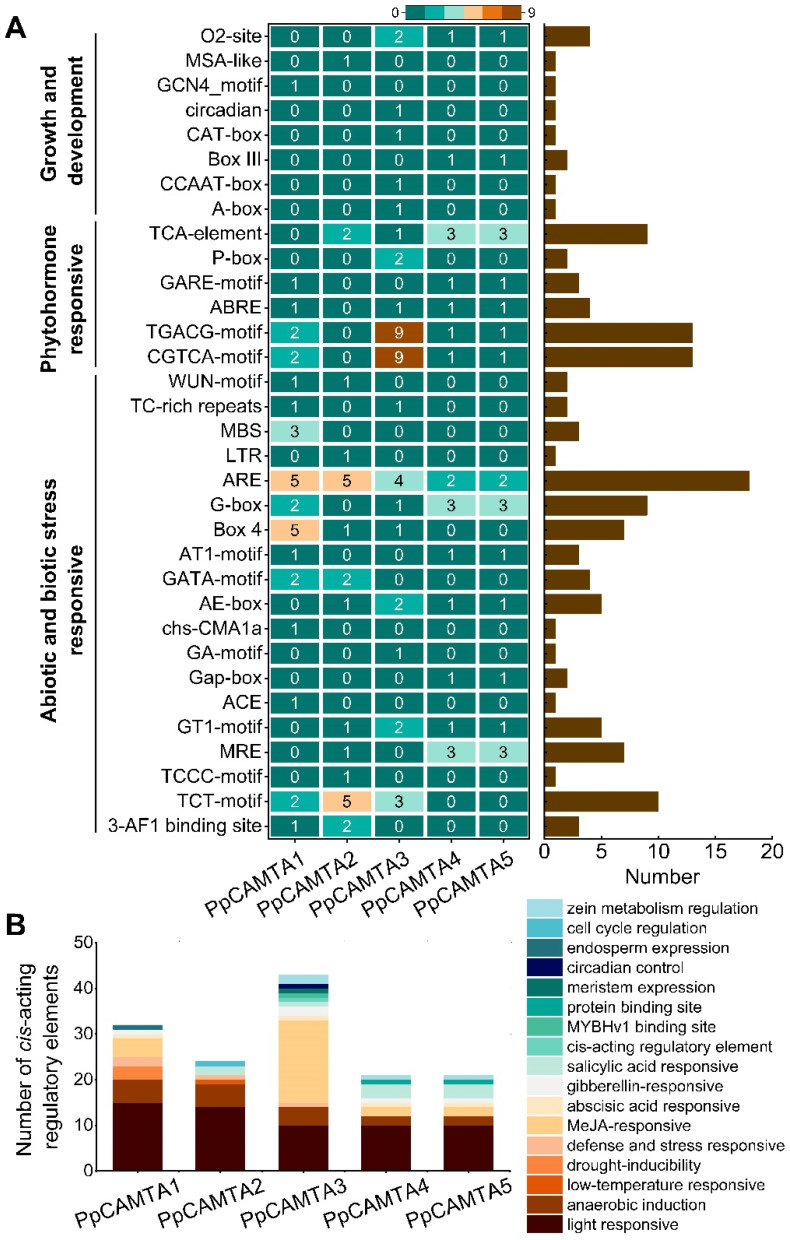
Analysis of *cis*-acting regulatory elements of peach *CAMTA*s. (**A**) Distribution of different *cis*-acting motifs for peach *CAMTAs* promoter region. Counts of each *cis*-acting motif were shown in the column diagram on the right. The *cis*-acting motifs were classified into three major groups depending on their functional annotation. (**B**) The number of predicted *cis*-acting elements in promoter region of peach *CAMTA* genes.

*Cis*-acting regulatory elements are specific motifs existing in the gene promoter regions, serve as binding sites for TFs to regulate gene transcription [34]. To gain more information about the response of peach *CAMTA* genes to stress and hormonal signals, *cis*-acting regulatory elements within 2000 bp upstream promoter region of peach *CAMTA* genes were predicted using the PlantCARE database. According to the annotations, we divided the predicted regulatory motifs into three categories: “growth and development”, “phytohormone responsive” and “abiotic and biotic responsive”. The promoters of peach *CAMTA* genes contained abundant stress-related and hormone-related regulatory motifs (Figure 3A). For instance, *PpCAMTA1* had TC-rich repeat and WUN-motif which respond to defense and stress.

The promoter regions of all peach *CAMTA* genes contained a large number of ‘light responsive’ motifs with 59 totally, including G-box, TCT motif, Box4, etc. Except for *AtCAMTA3*, the other four gene promoters had more than 50% regulatory motifs responding to light. Besides, the motifs involved in phytohormone responses were found in these promoters, including ABRE (abscisic acid-responsive elements), CGTCA-motif and TGACG-motif (methyl jasmonate-responsive elements), TCA-motif (salicylic acid-responsive elements) and P-box (gibberellin-responsive element) (Figure 3A). Especially for the *PpCAMTA3* promoter, the maximum number of ‘methyl jasmonate (MeJA) responsive’ motifs was found with 18. In addition, regulatory motifs that were critical for plant growth and development response were discovered in the promoters of peach *CAMTA* genes. Among these five peach *CAMTAs*, the promoter of *PpCAMTA3* has the largest number of regulatory motifs with 43, followed by *PpCAMTA1* and *PpCAMTA2* with 32 and 24, respectively (Figure 3B).

### 2.5. Expression of CAMTAs in Response to Cold, UV-B and MeJA Treatment in Peach Fruit

**Figure 4:**
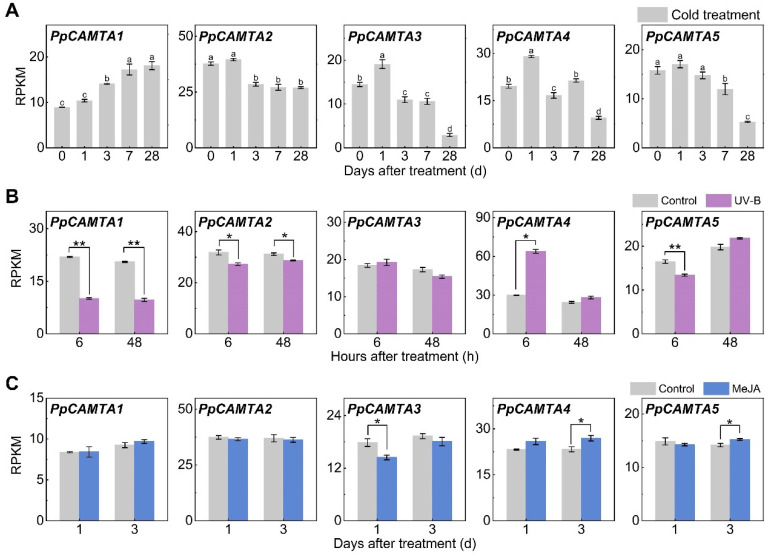
Expression patterns of peach *CAMTA* genes responding to abiotic stresses. (**A**) Cold treatment. (**B**) UV-B treatment. (**C**) MeJA treatment. Means and standard errors were calculated from three replicates. Different letters (*p* < 0.05) and asterisks (*, *p* < 0.05; **, *p* < 0.01) above the bars represent significant differences.

To explore the functional relevance of peach *CAMTA* genes, we next examined the expression patterns of *CAMTAs* under various treatments based on RNA-seq data. *PpCAMTA1* was significantly induced by low temperature (Figure 4A), the transcript level started increasing after 1-day cold treatment and kept rising until 28 days, about 2-fold higher than that of the initial point. On the contrary, the transcript levels of *PpCAMTA2* and *PpCAMTA5* were inhibited by low temperature (inhibited by 29 % and 67 % after 28 days, respectively). The expression of *PpCAMTA3* and *PpCAMTA4* were induced by low temperature and peaked at 1 d followed by a decline during cold storage.

Transcript levels of *PpCAMTA1* in UV-B treated group were reduced by approximately 50% relative to those of the control (Figure 4B). *PpCAMTA2* and *PpCAMTA5* were suppressed slightly by UV-B. In contrast, transcript level of *PpCAMTA4* in UV-B treated peach fruit was approximately 2-fold higher than that of control at 6 h, and decreased to the control level at 48 h. With regard to MeJA treatment, *PpCAMTA3* expression was inhibited by 20% at 1 d (Figure 4C). This was consistent with the rich MeJA-responsive motifs in *PpCAMTA3* promoter (Figure 3). *PpCAMTA4* and *PpCAMTA5* were slightly induced after being treated by MeJA for 3 days. Transcript levels of *PpCAMTA1* and *PpCAMTA2* remained unchanged after MeJA treatment (Figure 4C).

To confirm expression patterns of peach *CAMTAs* to various treatments, RT-qPCR analysis was performed. In agreement with RNA-seq results (Figure 4B), induced transcript levels of *PpCAMTA4* by UV-B were observed at 6 h as well (Figure S2A). UV-B treatment significantly reduced transcript levels of *PpCAMTA1* at both 6 h and 48 h (Figure 4B and Figure S2A). Regarding to MeJA treatment, transcript level of *PpCAMTA3* was reduced significantly at 1 d (Figure S2B), in agreement with RNA-seq results (Figure 4C). Significant induction of *PpCAMTA4* expression was detected at 3 d after MeJA treatment for both RNA-seq and RT-qPCR (Figure 4C and Figure S2B). Overall, similar gene expression patterns were observed between RT-qPCR and RNA-seq. Our results indicated that peach fruit *CAMTAs* responded to various stimuli in a different manner.

### 2.6. Expression of CAMTAs in Different Organs and During Fruit Development

**Figure 5:**
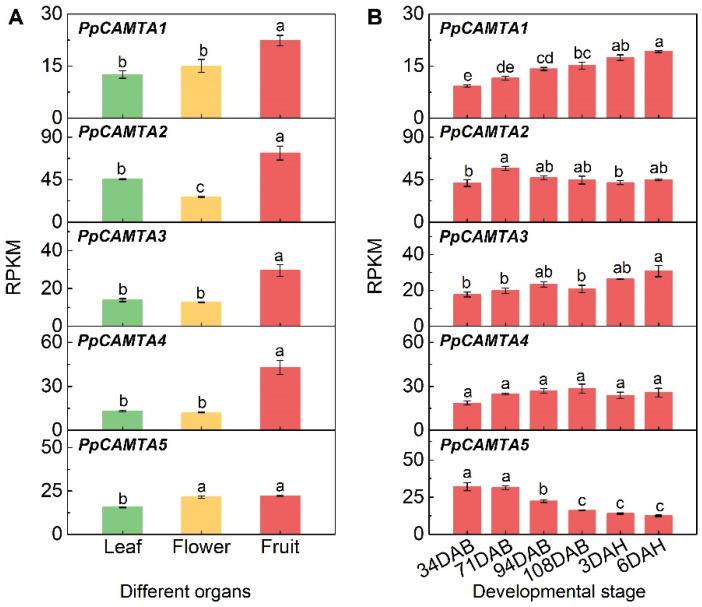
Expression of peach *CAMTA* genes in different organs and during fruit different stage. (**A**) Expression pattern of peach *CAMTA* genes in different organs including leaf (green), flower (yellow), and ripe peach fruit (red). (**B**) Expression pattern of peach *CAMTA* genes of different stages in peach fruit. Means and standard errors were calculated from three replicates. Different letters above the bars represent significant differences (*p* < 0.05).

To investigate the spatial and temporal expression patterns of peach CAMTA genes, their transcript levels in leaf, flower and fruit at different developmental stages were analyzed using RNA-seq. All peach *CAMTA* genes showed the highest transcript levels in ripening peach fruit comparing to leaf and flower (Figure 5A). Notably, the transcript level of *PpCAMTA4* in ripe fruit was 3.3 and 3.5-fold of that in leaf and flower, respectively. The expression of *PpCAMTA5* in flowers remained at the similar level as in ripe fruit. At the fruit development stages, *PpCAMTA1* was constantly accumulated with fruit ripening, peaked at 6 DAH when transcript level was about 2-fold higher than that at 34 DAB (Figure 5B). A similar pattern was observed for *PpCAMTA3*. The transcript level of *PpCAMTA2* increased slightly and reached the maximum at 71 DAB. The sustained decline was found in the transcript level of *PpCAMTA5*, which was inhibited by approximately 60% at 6 DAH compared with that at 34 DAB. Transcript levels of *PpCAMTA4* did not appear to be affected by fruit development. Taken together, peach *CAMTAs* exhibited fruit-specific and ripening-associated expression patterns, *PpCAMTA1* and *PpCAMTA3* were enriched in ripe fruit, suggesting that they may play important roles in peach fruit ripening.

### 2.7. Subcellular Localization of Peach CAMTA Proteins

**Figure 6:**
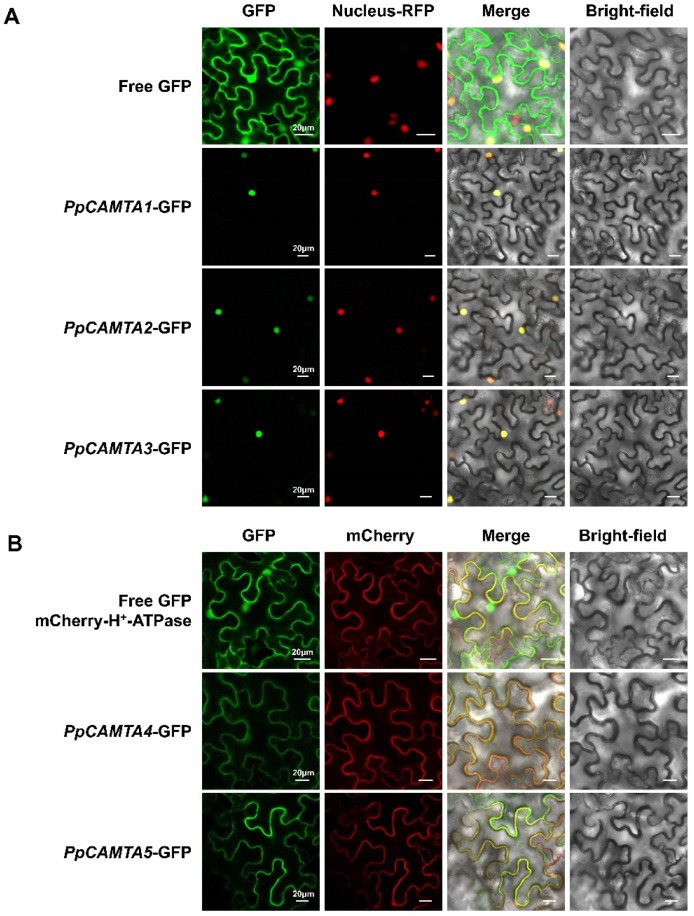
Subcellular localization of peach CAMTAs in *Nicotiana benthamiana* leaves. (**A**) Subcellular localization of PpCAMTA1, PpCAMTA2, PpCAMTA3. (**B**) Subcellular localization of PpCAMTA4, PpCAMTA5. GFP, GFP channel; Nucleus–RFP, transgenic tobacco with red fluorescence in the nucleus; mCherry-H^+^-ATPase, mCherry signal of H^+^-ATPase with red fluorescence as a marker in the plasma membrane; Merge, merged image of the GFP and nucleus–RFP or mCherry channels; Bright-field, light microscopy image; bars = 20 µm.

As CAMTA family members are transcription factors that contain the nuclear localization signal (NLS) domain, they were considered to be located in cell nucleus [23]. The NLS domains were predicted in peach CAMTA proteins (Figure 1B). In order to further understand the role of peach *CAMTA*s, subcellular localizations of these proteins were carried out by transiently expressing the *PpCAMTA*s-GFP constructs in tobacco leaves. For PpCAMTA1, PpCAMTA2 and PpCAMTA3, fluorescence microscopy showed distinct bright spots detected on the cell nucleus (Figure 6A), suggesting their nuclear localization. However, the green fluorescence from PpCAMTA4-GFP and PpCAMTA5-GFP proteins co-localized with the red fluorescence from mCherry-H^+^-ATPase in the plasma membrane of tobacco leaves (Figure 6B). This extranuclear localization of CAMTA proteins has also been reported in Arabidopsis [19]. Taken together, our results showed that PpCAMTA1, PpCAMTA2 and PpCAMTA3 were in the nucleus, PpCAMTA4 and PpCAMTA5 were located in the plasma membrane of the cell.

### 2.8. Overexpressing PpCAMTA1 Restored the Plant Size of Camta2,3 Mutant

The evolutionary tree has demonstrated the close phylogenetic relationships between peach CAMTA1/2/3 and Arabidopsis CAMTA1/2/3 (Figure 1A). In order to answer the question of whether PpCAMTA1/2/3 represents the functional homologs of AtCAMTA1/2/3, we expressed *PpCAMTA1*, *PpCAMTA2* and *PpCAMTA3* in the *camta2,3* mutant, respectively (Figure S3). We chose *camta2,3* as the genetic background for the same reasons as the previous study [15], *camta2,3* double mutant shows greater changes in SA biosynthesis and expression of SA pathway genes than those in the *camta3* single mutant, while *camta1,2,3* triple mutant is tiny in size and difficult to work with.

**Figure 7:**
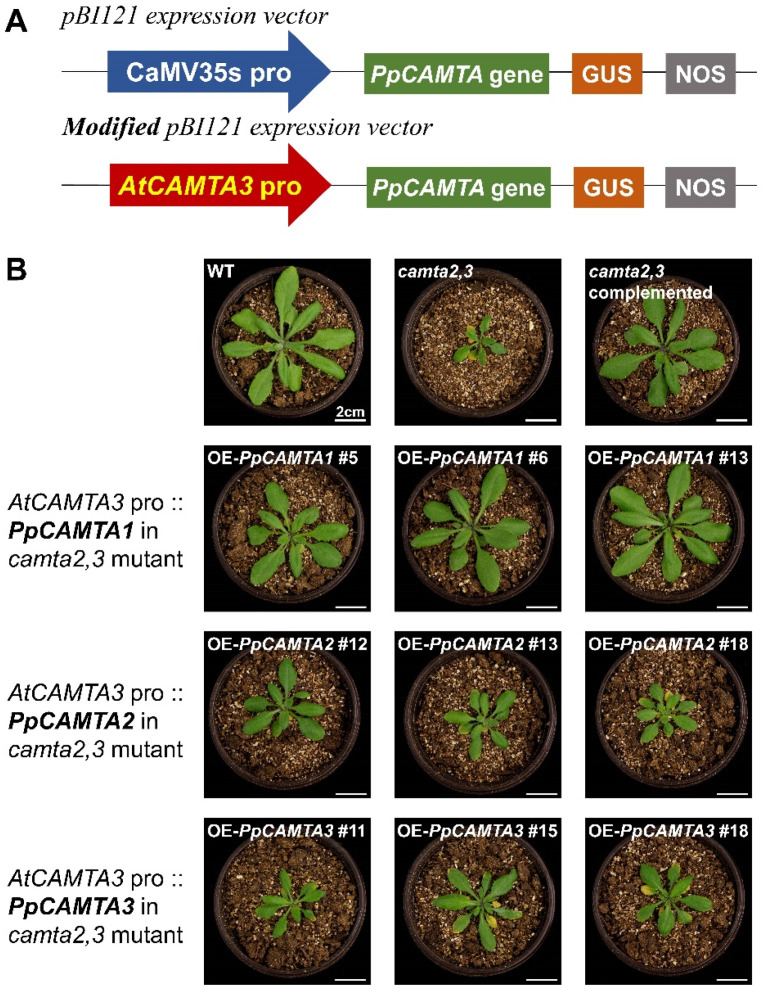
Overexpression of *PpCAMTA1/2/3* in Arabidopsis *camta2,3* mutant. (**A**) Schematic diagram of modified vector. (**B**) Photographs of the indicated plants grown at 22 °C for 28 days. WT: wild type; *camta2,3*: double mutant of *camta2* and *camta3*; OE*-PpCAMTA1*, OE*-PpCAMTA2* and OE*-PpCAMTA3*: overexpression of *PpCAMTA1, PpCAMTA2* and *PpCAMTA3* in the background of Arabidopsis *camta2,3* mutant driven by *AtCAMTA3* promoter, respectively.

To avoid the excessively strong expression caused by the CaMV35S promoter, we replaced the 35S promoter of the pBI121 vector with the endogenous promoter of *AtCAMTA3* to ensure the natural expression level (Figure 7A). The results showed that overexpression of these three peach *CAMTA* genes complemented the plant size of *camta2,3* to different extent. As shown in Figure 7B, the plant size of OE-*PpCAMTA1* transgenic lines were larger than *camta2,3* and closed to that of wild type (WT). Although OE-*PpCAMTA2* and OE-*PpCAMTA3* transgenic lines did not complement the plant size to the level of wild type, expression of these two genes did result in larger plant size than that of *camta2,3* mutant (Figure 7B). In brief, peach *PpCAMTA1* could largely rescue the plant size defect phenotype caused by *camta2,3* mutation, *PpCAMTA2* and *PpCAMTA3* partially restored plant development after overexpressing in *camta2,3* mutant.

### 2.9. Overexpressing PpCAMTA1 Reduces SA Biosynthesis and Weakens Plant Resistance to Pathogen of Camta2,3 Mutant

Our results showed that overexpressing *PpCAMTA1* recovered *camta2,3* mutant size close to that of WT (Figure 7B), indicating that *PpCAMTA1* is a candidate gene with a role similar to Arabidopsis *CAMTA3* during plant development. Arabidopsis CAMTA1, CAMTA2 and CAMTA3 repress SA biosynthesis and *ICS1* expression by regulating transcription factors at ambient temperature [14,17]. In addition, CAMTA3 regulates *SARD1* and *CBP60g* expression [18]. Therefore, we next explored the role of *PpCAMTA1* in plant immune response.

**Figure 8:**
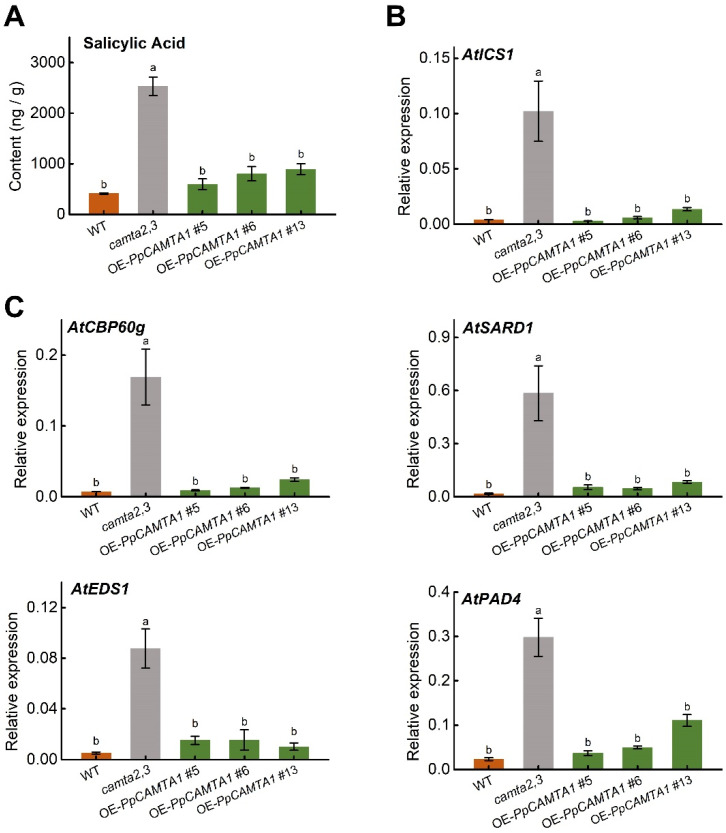
Overexpression of *PpCAMTA1* in *camta2,3* mutants inhibited SA level and SA pathway genes expression levels. (**A**) Free SA content in 4-week-old wild type, *camta2,3* and OE-*PpCAMTA1* plants. (**B**) Transcript levels for SA synthesis related gene *AtICS1*. (**C**) Transcript levels for SA pathway transcription factors. Means and standard errors were calculated from three replicates. Different letters above the bars represent significant differences (*p* < 0.05).

Significantly higher SA content and related genes expression levels were observed in *camta2,3* mutant than those in WT (Figure 8). After overexpressing *PpCAMTA1*, transgenic plants resulted in at least 52 % reduction in SA content in relative to that of *camta2,3* mutant (Figure 8A). Correspondingly, transcript levels of *AtICS1* (a gene responsible for SA synthesis) were reduced by approximately 90% in OE-*PpCAMTA1* lines when compared to that in *camta2,3* mutant (Figure 8B). Besides SA content and SA synthesis related gene expression, expression levels for SA related transcription factors were analyzed as well. Transcription levels of *AtCBP60g* in transgenic lines were about 5.3%, 7.3% and 14.2% of that in *camta2,3*, respectively (Figure 8C). Moreover, in transgenic lines after overexpressing *PpCAMTA1*, expression levels of *AtSARD1*, *AtEDS1* and *AtPAD4* were significantly lower than those in *camta2,3* mutant as well. It is noteworthy that both SA content and expression levels for genes associated with SA synthesis and signaling were similar between WT and transgenic plants after overexpressing *PpCAMTA1*.

**Figure 9:**
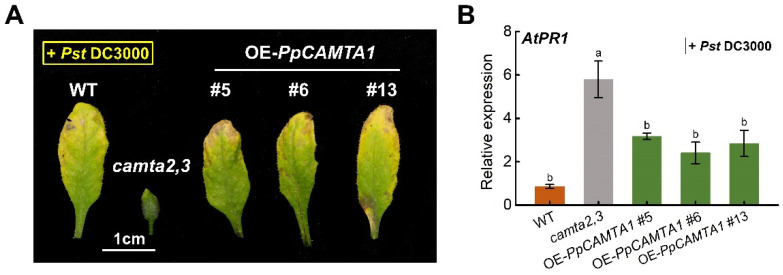
Overexpression of *PpCAMTA1* in *camta2,3* mutant affects plant response to *Pst* DC3000. (**A**) Photos of 4-week-old plants leaves at 3 days post-inoculation (3 dpi) with the spray of *Pst* DC3000. (**B**) Transcript level of *PR1* after 1 dpi with the spray of *Pst* DC3000. Means and standard errors were calculated from three replicates. Different letters above the bars represent significant differences (*p* < 0.05).

Given the above phenotype, *Pst* DC3000 (*Pseudomonas syringae* pv. tomato DC3000) was used to measure the effect of *PpCAMTA1* on pathogen resistance. Three days after pathogen infection, *camta2,3* mutant plants exhibited scarce chlorotic spots on their leaves, while wild type and OE-*PpCAMTA1* lines exhibited severity chlorotic spots on leaves, showed more susceptibility to the *Pst* strain (Figure 9A). Gene expression was also shown to induce stronger *AtPR1* in *camta2,3* mutants after 1 d of inoculation when compared with OE-*PpCAMTA1* lines (Figure 9B). The expression of SA-related genes mentioned above showed similar trends among these plants (Table S6). Taken together, these results revealed that *PpCAMTA1* negatively regulated SA biosynthesis and genes related to SA pathway, which may act as a negative regulator of plant immunity in peach.

## 3. Discussion

As important transcription factors containing CaM binding sites, the functions of *CAMTAs* have been reported widely in recent years [7,23,35]. Although comprehensive genome-wide analysis of *CAMTAs* have been reported in horticultural crops [26,27,28,29], functions of *CAMTAs* in fruit remain largely unknown. Peach is considered as a model plant of *Rosaceae* family [31]. In this study, we identified five peach *CAMTA* genes. Similar number of CAMTA genes were also observed for sweet cherry, apricot and almond. *Rosaceae* family fruit crops such as apple and strawberry have more CAMTA gene members. The number of CAMTA genes varing in different plant species is suggested to be associated with evolution although the process is complex [7].

Pathogen infection and disease development are major factors that cause postharvest decay, leading to a limitation in the storage period and marketing life of peach fruit [36]. Improving immunity resistance is an efficient and preferred strategy for reducing disease development in fruit [37]. Several studies have shown that application of calcium before and after harvesting delayed the development of peach fruit disease, and thus prolonged storage period of peach fruit [38,39,40]. Therefore, it is necessary to identify fruit immune-related transcription factors on calcium signaling pathways. In Arabidopsis, AtCAMTA1/2/3 are components derived from calcium signaling pathway and play negative roles in plant immune responses. For instance, mutant *camta2,3* has increased SA content and higher expression levels of SA-regulated immunity genes than those in wild type Arabidopsis [14]. Overexpressing *PpCAMTA1* in the *camta2,3* mutants resulted in lower SA content, contributing to more susceptible to the virulent *Pst* DC3000 strain. Notably, overexpression of *PpCAMTA1* restored plant size, close to that of WT plant. These results indicated that *PpCAMTA1* could functionally complement Arabidopsis *CAMTA2/3* by overexpressing peach gene in *cmata2,3* mutant. During peach fruit storage at ambient temperature, transcript levels of *PpCAMTA1* increased, accompanied with fruit postharvest ripening and senescence. Similarly, increased expression levels were also observed for tomato *SlSR1/1L* [10] and durian *DzCAMTA3*/*8* during postharvest fruit ripening [30]. Induced expression of tobacco *CAMTA* member *NtER1* was suggested to be associated with ethylene induced senescence for both leaf and flower [6]. According to expression pattern of *PpCAMTA1* and transgenic Arabidopsis phenotype in *camta2,3* genetic background, our results suggested that increased *PpCAMTA1* expression was associated with reduced peach fruit immune response during postharvest storage, thus contributing to fruit senescence and seed dispersal.

Apart from biological stress, expression of plant *CAMTAs* could also be affected by abiotic stresses [23]. However, effect of abiotic stresses on expression of *CAMTAs* has not been well studied in fruit species. In our study, we investigated peach *CAMTA* genes expression profiles during peach fruit ripening and in response to stresses. The present study revealed that *PpCAMTA1* expression was induced by cold treatment in peach fruit, while the expression of the left four members was inhibited during postharvest cold storage for 28 d. In tomato fruit, transcript levels of *SlSR2* and *SlSR3L* were induced by cold treatment [10]. Transcript levels of Arabidopsis *AtCAMTA3* increased and stayed at high level during exposure to low temperature for 5 weeks [14]. These results showed that induced expression of *CAMTAs* was conserved both in plants and in fruit species, indicating an important role of peach *PpCAMTA1* during fruit cold storage. UV-B irradiation for Arabidopsis seedlings induced expression of *CAMTAs* [41]. Here, we showed that expression levels of *PpCAMTA1*, *PpCAMTA2* and *PpCAMTA5* were inhibited by UV-B. Based on RT-qPCR results, expression level of *PpCAMTA3* was also repressed by UV-B (Figure S2A). These results revealed different response of *CAMTA* expression patterns between Arabidopsis leaf and peach fruit upon to UV-B treatment. Our previous study showed that UV-B had great effects on contents of both anthocyanin and volatile linalool in peach fruit [42,43], suggesting that peach *CAMTAs* is associated with the formation of fruit secondary metabolites.

Most previous reports showed that CAMTAs belong to nuclear protein [9,44]. Arabidopsis CAMTA2, CAMTA3 and CAMTA5 were located in nuclei [19]. The present study showed that peach CAMTAs were located both in nucleus and plasma membrane. For instance, PpCAMTA1, PpCAMTA2 and PpCAMTA3 were located in nucleus, and PpCAMTA4 and PpCAMTA5 were located in the plasma membrane. Phylogenetic analysis showed that PpCAMTA4 and PpCAMTA5 were on different clades from PpCAMTA1-3 due to difference in sequence. A previous study analyzed the subcellular localization of 465 CAMTA proteins, of which 390 proteins were predicted to located in the nucleus, and the remaining CAMTAs were localized in the cytosol, chloroplast and plasma membrane [7]. Some CAMTAs contain nuclear localization signal sequence which allows these proteins to enter the nucleus to perform their functions. Moreover, these extranuclear localizations of CAMTAs have also been reported in Arabidopsis. For example, destabilization and nuclear export were observed for AtCAMTA3 after protein phosphorylation modification caused by mitogen-activated protein kinases (MAPKs) MPK3 and MPK6 [45]. These two MAPKs were induced by bacterial flg22 elicitor in Arabidopsis. Interestingly, Arabidopsis AtCAMTA1, AtCAMTA4, and AtCAMTA6 were suggested to be unstable at room temperature or 4 °C [19]. These results indicated that it is not surprising that PpCAMTA4/5 were located outside the nucleus. The mechanisms need to be further investigated for the different location of PpCAMTAs in future.

Among five peach CAMTA gene members, PpCAMTA1, PpCAMTA2 and PpCAMPT3 were grouped with Arabidopsis AtCAMTA3 which is involved in plant development. To test if peach CAMTA genes could functionally complement the defect phenotype in Arabidopsis mutant, *PpCAMTA1*, *PpCAMTA2* and *PpCAMPT3* were overexpressed driven by the native *AtCAMTA3* promoters. The second reason to choose the native *AtCAMTA3* promoter is to avoid the excessively strong expression caused by the CaMV35S promoter. We found that overexpression of these three peach *CAMTA* genes complemented the plant size of *camta2,3* to different extent. Peach *PpCAMTA1* rescued the developmental defect phenotype caused by *camta2,3* mutation, restored the plant size to the WT level. Notably, only partially restored plant development phenotype was observed for *PpCAMTA2* and *PpCAMTA3*. However, we cannot exclude roles of other peach *CAMTA* genes during plant development. The identification of functions of each peach CAMTA gene will require further research. Our dataset in hand suggest that *PpCAMTA1* is associated with plant development, including fruit ripening and postharvest responses to biotic and abiotic stresses.

## 4. Materials and Methods

### 4.1. Plant Material and Treatment

Peach (*Prunus persica* L. Batsch cv. Hujingmilu) fruit at different developmental stages, fully expanded leaves and flowers at bloom stage were used for gene expression analysis according to our previous study [46]. After harvest at mature stage (108 days after bloom, DAB), peach fruits were held at 20 °C for 3 d (3 days after harvest, DAH) and 6 d (6 DAH). Treatments with UV-B irradiation [42] and methyl jasmonate (MeJA) [47] were used in this study. For cold treatment, peach fruits were stored at 0 °C up to 28 days. Three biological replicates with five fruits each were sampled, frozen in liquid nitrogen immediately and stored at −80 °C for further analysis.

### 4.2. Identification of Peach CAMTA Genes

The peach *CAMTA* genes were identified by the following approaches. Firstly, searching in the peach genome database at Phytozome v12.1 website (https://phytozome.jgi.doe.gov, accessed on 24 February 2022) based on gene annotations. Secondly, sequences of Arabidopsis CAMTA members were used as queries to search against the peach genome database with BLASTN and BLASTP programs. Thirdly, sequences of peach CAMTAs were checked for CAMTA conserved protein domains by using HMMER (http://www.ebi.ac.uk, accessed on 24 February 2022), Pfam database (http://pfam.janelia.org/, accessed on 24 February 2022) and SMART (http://smart.embl-heidelberg.de/, accessed on 24 February 2022). Peach *CAMTA* genes were cloned and sequenced for the subsequent analysis. Finally, sequence information of *CAMTA* genes was also extended to various genome assemblies in *Prunus persica* and *Rosaceae* genome whose sequence were gained from GDR website (https://www.rosaceae.org/, accessed on 22 August 2022).

### 4.3. Physicochemical Property Analysis and Phylogenetic Analysis

Amino acid properties and physicochemical traits of the given peach CAMTA proteins were calculated using the ProtParam tool in the ExPASy web server (https://web.expasy.org/protparam/, accessed on 28 February 2022). The full-length amino acid sequences of CAMTAs in Arabidopsis and peach were used to perform the multiple sequence alignments using Clustal W with default parameters. The phylogenetic analysis was constructed using the neighbor-joining (NJ) method with 1000 bootstrap replications by MEGA 11. Peach *CAMTA* genes were named based on the genetic relationship with Arabidopsis CAMTA genes.

### 4.4. Exon-Intron Organization and Chromosomal Map Construction

The distribution patterns of exon-intron of peach *CAMTA* genes were obtained from the peach genome database and displayed using the online website Gene Structure Display Server v2.0 (GSDS, http://gsds.cbi.pku.edu.cn/, accessed on 11 March 2022). The locations of *CAMTA* genes on the chromosomes were visualized by MapChart (v2.3) (Voorrips, R.E., Wageningen, The Netherlands).

### 4.5. Conserved Protein Domains Distribution and Cis-Regulatory Element Analysis

Bioinformatic analysis of the conserved protein domains was conducted through the online SMART tool (http://smart.embl-heidelberg.de/, accessed on 28 February 2022). CaM binding domain (CaMBD) was predicted by the Calmodulin Target Database (http://calcium.uhnres.utoronto.ca/ctdb/ctdb/, accessed on 28 February 2022). A schematic representation of conserved protein domain structures was constructed using DOG 2.0 software (Jian Ren, Hefei, China) [48]. Online server MEME (Multiple Em for Motif Elicitation) was used to carry out motif analysis. For promoter *cis*-regulatory elements analysis, putative promoter sequences (2 kb from TSS) of the peach *CAMTA* genes were downloaded from the peach genome database, and then identification of possible *cis*-acting regulatory elements was performed by PlantCARE (http://bioinformatics.psb.ugent.be/webtools/plantcare/html/, accessed on 11 March 2022).

### 4.6. Subcellular Localization Analysis

Subcellular localization analysis was conducted according to our previous study [49]. The recombined 35S-*PpCAMTA*-eGFP vectors were constructed using primers listed in Table S3. To identify the nuclear location, the vector was infiltrated into transgenic tobacco plants expressing a red fluorescent nuclear marker (Nucleus–RFP). To verify the plasma membrane localization, mCherry-H^+^-ATPase with red fluorescence was used as a marker of plasma membrane [50]. Leaves were detached for analysis using a confocal laser scanning microscope (LSM 780; Carl Zeiss, Oberkochen, Germany).

### 4.7. RNA Extraction and Gene Expression Analysis

Total RNA of peach fruit was extracted according to our previous study [51], and libraries for high-throughput Illumina strand-specific RNA-sequencing were prepared as described previously [52]. Reads per kilobase of exon model per million mapped reads (RPKM) based on the length of the gene and the number of reads mapped to this gene was used to express transcript abundance [42,46]. Three biological replicates for each sampling time point were performed for RNA-Seq.

Total RNA of Arabidopsis plant leaves was extracted by using the reagent RNAiso Plus (Takara) according to its instructions. For quantitative reverse-transcription PCR (RT-qPCR) analysis, HiScript II Q RT SuperMix for qPCR (+gDNA wiper) and ChamQ Universal SYBR qPCR Master Mix (Vazyme, Nanjing, China) were used. Each RT-qPCR analysis contained three biological replicates. Oligonucleotide primers for RT-qPCR analysis were listed in Table S4.

### 4.8. Stable Overexpression of Arabidopsis

The transformation was conducted in four-week-old seedlings of Arabidopsis plants according to the floral dip method. The *camta2,3* double mutant T-DNA insertion mutants were in Col-0 background and got from Kim [14]. The modified pBI121-*PpCAMTA1/2/3* constructs were used to obtain overexpression (OE) lines in the *camta2,3* genetic background. Notably, the overexpression of peach *PpCAMTA1/2/3* was driven by the *AtCAMTA3* promoter rather than the CaMV35S promoter. Primers were listed in Table S5. Transgenic seeds were selected on 1/2 Murashige and Skoog (MS) medium with 50 mg/L kanamycin. Wild type (WT) and mutants were planted in a growth chamber with 22 °C under 16 h light (100 μmol m^−2^ s^−1^ constant light) and 8 h dark. After 1-month growth, rosette-like basal leaves of the above-ground part were sampled for subsequent analysis.

### 4.9. Pathogen Infection Assay

For infection with *Pst* DC3000 (*Pseudomonas syringae* pv. tomato DC3000), some modifications were made by referring to the methods of previous studies [18]. Briefly, leaves of 4-week-old plants were sprayed with the bacteria at a dose of OD_600_ = 0.002 in 10 mM MgCl_2_. Infected leaves were covered with a clean dome to maintain high humidity, grown at 22 °C under 16/8-h light/dark cycles in a growth chamber. On 1 day after inoculation, the leaf samples were collected for RNA extraction and gene expression analysis, and plants scored 3 days for phenotypic observation.

### 4.10. Quantification of Salicylic Acid (SA) Content

Free SA content was extracted and measured using the previous methods with some modifications [53]. A total of 0.2 g fresh weight of leaf tissue powder was homogenized and extracted for 4 h at 65 °C using 1.2 mL of 90% (*v*/*v*) methanol. After centrifugation, the supernatants were dried in a speed vacuum at 60 °C. The residue was resuspended using 600 μL 5% (*v*/*v*) trichloroacetic acid and sonicated for 10 min. Then, 2 vol (1.2 mL) of ethylacetate-cyclopentane-isopropanol (50:50:1) were added to extract SA. The superorganic phase containing the SA was dried in a speed vacuum with 60 °C. The residue was dissolved in 500 μL methanol. SA was detected using a fluorescence detector in HPLC analysis, the excitation wavelength is 295-nm, and the emission wavelength is 405-nm.

### 4.11. Statistical Analysis

A completely randomized design was used in the experiments. Mean value and standard errors (SE) were calculated by Microsoft Excel, figures were produced by OriginPro 9 (Microcal Software Inc., Northampton, MA, USA). The two-sample significance test was calculated using unpaired Student’s *t*-test (*, *p* < 0.05 and **, *p* < 0.01). For multiple samples significance test, ANOVA analysis followed by a post-hoc Tukey HSD test was used to calculate *p* value, and letters were designated for significantly different values (SPSS 19.0; SPSS Inc., Chicago, IL, USA).

## 5. Conclusions

In this study, a total of five CAMTA genes were identifed in peach (*Prunus persica* L. Batsch) at the level of gene structure, sequence characteristics, promoter cis-acting elements and expression patterns. Increased transcription levels were detected for *PpCAMTA1* during fruit development and ripening. Our hypothesis that PpCAMTA1 is an ortholog of AtCAMTA3 was experimentally confirmed by overexpressing *PpCAMTA1* under the control of the native *AtCAMTA3* promoter in Arabidopsis mutant system. Overexpressing *PpCAMTA1* can complement the developmental deficiencies in Arabidopsis *camta2,3*, indicating an association of PpCAMTA1 during developmental process such as fruit ripening and postharvest responses to biotic and abiotic stresses.

## Figures and Tables

**Table 1:** Physicochemical properties of peach CAMTA proteins.

Gene Name	Locus ID	Length (aa)	MW (kD)	pI	II	Aliphatic Index	GRAVY
*PpCAMTA1*	Prupe.1G108700	1012	113.28	6.69	42.20	76.91	−0.522
*PpCAMTA2*	Prupe.8G060300	1086	121.55	5.69	44.84	78.84	−0.514
*PpCAMTA3*	Prupe.6G187700	1131	126.59	5.69	39.54	77.29	−0.540
*PpCAMTA4*	Prupe.1G224000	994	110.91	5.58	44.52	72.61	−0.578
*PpCAMTA5*	Prupe.1G122800	914	103.20	6.62	44.54	84.32	−0.422

Notes: Grand average of hydropathicity (GRAVY); Instability index (II).

**Table 2:** The number of CAMTA genes in fruit crops from *Rosaceae* family.

Fruit Name	Organism	Number of CAMTAs
Strawberry	*Fragaria x ananassa*	16
Pear	*Pyrus pyrifolia*	10
Apple	*Malus domestica*	8
Sweet cherry	*Prunus avium*	5
Almond	*Prunus dulcis*	5
Peach	*Prunus persica*	5
Apricot	*Prunus armeniaca*	4

## Data Availability

The relevant datasets can be found in the NCBI with accession number PRJNA576753, PRJNA574004, PRJNA852345 and SRP103523.

## Supplementary Materials

The following supporting information can be downloaded at: https://www.mdpi.com/article/10.3390/ijms231810500/s1. References [54,55,56,57,58] are cited in the Supplementary Materials.
