# Supplementary material for: Genome-Wide Analysis of Calmodulin Binding Transcription Activator (CAMTA) Gene Family in Peach (Prunus persica L. Batsch) and Ectopic Expression of PpCAMTA1 in Arabidopsis camta2,3 Mutant Restore Plant Development
Source: Int J Mol Sci. 2022 Sep 10;23(18):10500. doi: 10.3390/ijms231810500 (PMC9499639; doi:10.3390/ijms231810500)

Supplementary Material

Table S1. CAMTA genes of different genome assemblies in *Prunus persica*.

Peach varieties	*Prunus persica* Genome	Gene ID	Genome references
'Lovell'	Whole Genome Assembly v2.0	Prupe.1G108700.1<br>Prupe.8G060300.1<br>Prupe.6G187700.1<br>Prupe.1G224000.1<br>Prupe.1G122800.1	54, 55
'Chinese Cling'	Chinese Cling Genome V1.0	evm.model.contig279.973<br>evm.model.contig125.12<br>evm.model.contig7.29<br>evm.model.contig275.159<br>evm.model.contig279.1102	56
'Zhongyoutao 14'	Zhongyoutao 14 Genome v1.0	Pp01G011310.1<br>Pp08G008580.1<br>Pp06G017540.1<br>Pp01G028410.1<br>Pp01G012670.1	57
'124 Pan'	124 Pan Genome v1.0	P124PAN14929.1<br>P124PAN03468.1<br>P124PAN21190.1<br>P124PAN16004.1<br>P124PAN15065.1	58

References listed in the main text.

Table S2. Fruit CAMTA genes in *Rosaceae* family.

Fruit name	Organism	Genome source	Gene number	Gene ID	Length (aa)
Strawberry	*Fragaria x ananassa*	Genome v1.0	16	FxaC_13g22180.t2	1004
				FxaC_13g26730.t1	914
				FxaC_13g38580.t1	907
				FxaC_14g14610.t1	1005
				FxaC_14g18980.t1	928
				FxaC_15g15470.t1	1005
				FxaC_17g39580.t1	989
				FxaC_18g15530.t1	989
				FxaC_19g28040.t1	987
				FxaC_1g24361.t1	1074
				FxaC_20g31520.t1	987
				FxaC_2g28010.t1	1073
				FxaC_3g23640.t1	654
				FxaC_3g23862.t1	879
				FxaC_3g23863.t1	1074
				FxaC_4g16590.t1	1191
Apple	*Malus domestica*	Genome v1.0	8	MD01G1031100	1107
				MD05G1044600	1070
				MD10G1051700	1074
				MD13G1121200	977
				MD13G1144300	1004
				MD15G1318400	1108
				MD16G1121200	977
				MD16G1157900	910
Pear	*Pyrus pyrifolia*	Genome v1.0	10	Ppy00g1083.1	1125
				Ppy01g0314.1	1111
				Ppy05g0286.1	1075
				Ppy13g0414.1	1176
				Ppy13g1287.1	982
				Ppy13g1472.1	1010
				Ppy13g1558.1	916
				Ppy15g2682.1	1114
				Ppy16g0935.1	987
				Ppy16g1031.1	881
Sweet cherry	*Prunus avium*	Genome v1.0	5	Pav_sc0000028.1_g850.1.mk	1027
				Pav_sc0000091.1_g250.1.mk	1100
				Pav_sc0000334.1_g570.1.mk	1138
				Pav_sc0001031.1_g010.1.mk	914
				Pav_sc0010200.1_g030.1.mk	993
Apricot	*Prunus armeniaca*	Genome v1.0	4	PARG04349m01	1268
				PARG04567m01	898
				PARG05795m01	994
				PARG28696m01	1086

Almond	*Prunus dulcis*	Genome v2.0	5	Prudul26A007803	1131
				Prudul26A015191	914
				Prudul26A016773	1086
				Prudul26A019053	1085
				Prudul26A030701	894

Table S3. Primer sequences for 35S-*PpCAMTAs*-GFP vectors recombination.

Primers	Sequence (5′→3′)
eGFP-*PpCAMTA1*-FP	ctgcccaaattcgcgaccggtATGGCTGATACCAGAAAATACCTCC
eGFP-*PpCAMTA1*-RP	tcctttgctagtcataccggtTTGACCTTCGGTGAGTGCAAG
eGFP-*PpCAMTA2*-FP	ctgcccaaattcgcgaccggtATGGCGGAACGCGTATCG
eGFP-*PpCAMTA2*-RP	tcctttgctagtcataccggtATCAAATGCTATAGACATGAAAGTGTCG
eGFP-*PpCAMTA3*-FP	ctgcccaaattcgcgaccggtATGTATGAGATACAAGTTGGCTCTGG
eGFP-*PpCAMTA3*-RP	tcctttgctagtcataccggtTGGACCTGCTGTAGGCATGAA
eGFP-*PpCAMTA4*-FP	ctgcccaaattcgcgaccggtATGATGCAATCAGGGTATAATATTAATG
eGFP-*PpCAMTA4*-RP	tcctttgctagtcataccggtGGGAAACTGATACATATCAATGTCTTC
eGFP-*PpCAMTA5*-FP	ctgcccaaattcgcgaccggtATGGAAAAGCAGCTTGGGG
eGFP-*PpCAMTA5*-RP	tcctttgctagtcataccggtACTATCCATGTTAGAGTCAGGATCCA

Lowercase letters indicate sequences on the vector.

Table S4. Primer sequences for RT-qPCR analysis.

Primers	Sequence (5′→3′)
qPCR-*PpCAMTA1*-FP	ACCTAATTGCACGTCGTTCC
qPCR-*PpCAMTA1*-RP	TTCTTCCGAACCTGATGTCC
qPCR-*PpCAMTA2*-FP	AAATCCAGGCTCATGTACGG
qPCR-*PpCAMTA2*-RP	CAGTCCCCTTACGTCTCCAA
qPCR-*PpCAMTA3*-FP	CAGCCATTCCTGGGGTGAAT
qPCR-*PpCAMTA3*-RP	CAGCCTCCCATAGCGAAGAG
qPCR-*AtICS1*-FP	TATCTCCGGCAGCCGCCACT
qPCR-*AtICS1*-RP	ACGCCGGAGGAAAACGACGG
qPCR-*AtEDS1*-FP	CGAGACACATCAACTGTTGCAA
qPCR-*AtEDS1*-RP	CGCCTCTGCGCTTCCA
qPCR-*AtSARD1*-FP	TCGAGTTGGATTCGTAGCCG
qPCR-*AtSARD1*-RP	TCGCTTCAGTCATCGCTTCA
qPCR-*AtCBP60g*-FP	TCGTGGACGCCACCACAAACA
qPCR-*AtCBP60g*-RP	TCAGCGTTCAGCGGCACGAG
qPCR-*AtPAD4*-FP	ATCTTCTCCGCCGTCATTCC
qPCR-*AtPAD4*-RP	CACGTGGCAGAAGTTGTGTG
qPCR-*AtPR1*-FP	CACATCCGAGTCTCACTGAC
qPCR-*AtPR1*-RP	CAGACTCATACACTCTGGTG
AtEF1α-FP	TGAGCACGCTCTTCTTGCTTTCA
AtEF1α-RP	GGTGGTGGCATCCATCTTGTTACA

Table S5. Primer sequences for *AtCAMTA3* pro::*PpCAMTA*-pBI121 vectors recombination.

Primers	Sequence (5′→3′)
pBI121-*PpCAMTA1*-FP	cggagaaagcaatattctagaATGGCTGATACCAGAAAATA
pBI121-*PpCAMTA1*-RP	ggactgaccacccggggatccTTGACCTTCGGTGAGTGCAA
pBI121-*PpCAMTA2*-FP	cggagaaagcaatattctagaATGGCGGAACGCGTATCGTA
pBI121-*PpCAMTA2*-RP	ggactgaccacccggggatccATCAAATGCTATAGACATGA
pBI121-*PpCAMTA3*-FP	cggagaaagcaatattctagaATGTATGAGATACAAGTTGG
pBI121-*PpCAMTA3*-RP	ggactgaccacccggggatccTGGACCTGCTGTAGGCATGA
AtCAMTA3 promoter-FP	CTTTTTCCTTTTACTATCTTATTCTCTATCT
AtCAMTA3 promoter-RP	GATATTGCTTTCTCCGATCTCAGAG

Lowercase letters indicate sequences on the vector.

Table S6. Transcription levels for SA-related genes after 1 day infected with *Pst* DC3000. Means and standard errors were calculated from three replicates, different letters represent significant differences ($P < 0.05$).

Gene Name	WT	*camta2,3*	OE-*PpCAMTA1*		
			#5	#6	#13
AtEDS1	0.033±0.002a	0.042±0.003a	0.035±0.003a	0.038±0.004a	0.047±0.009a
AtPAD4	0.038±0.005b	0.078±0.009a	0.058±0.004ab	0.051±0.002b	0.060±0.003ab
AtSARD1	0.043±0.007b	0.117±0.013a	0.071±0.006ab	0.070±0.017ab	0.099±0.020ab
AtCBP60g	0.017±0.003a	0.036±0.006a	0.032±0.004a	0.025±0.005a	0.029±0.006a
AtICS1	0.016±0.004a	0.031±0.004a	0.021±0.002a	0.028±0.008a	0.029±0.005a

Figure S1. Conserved motifs in *CAMTAs* were identified using MEME suite program. Each motif is displayed with a specific color.

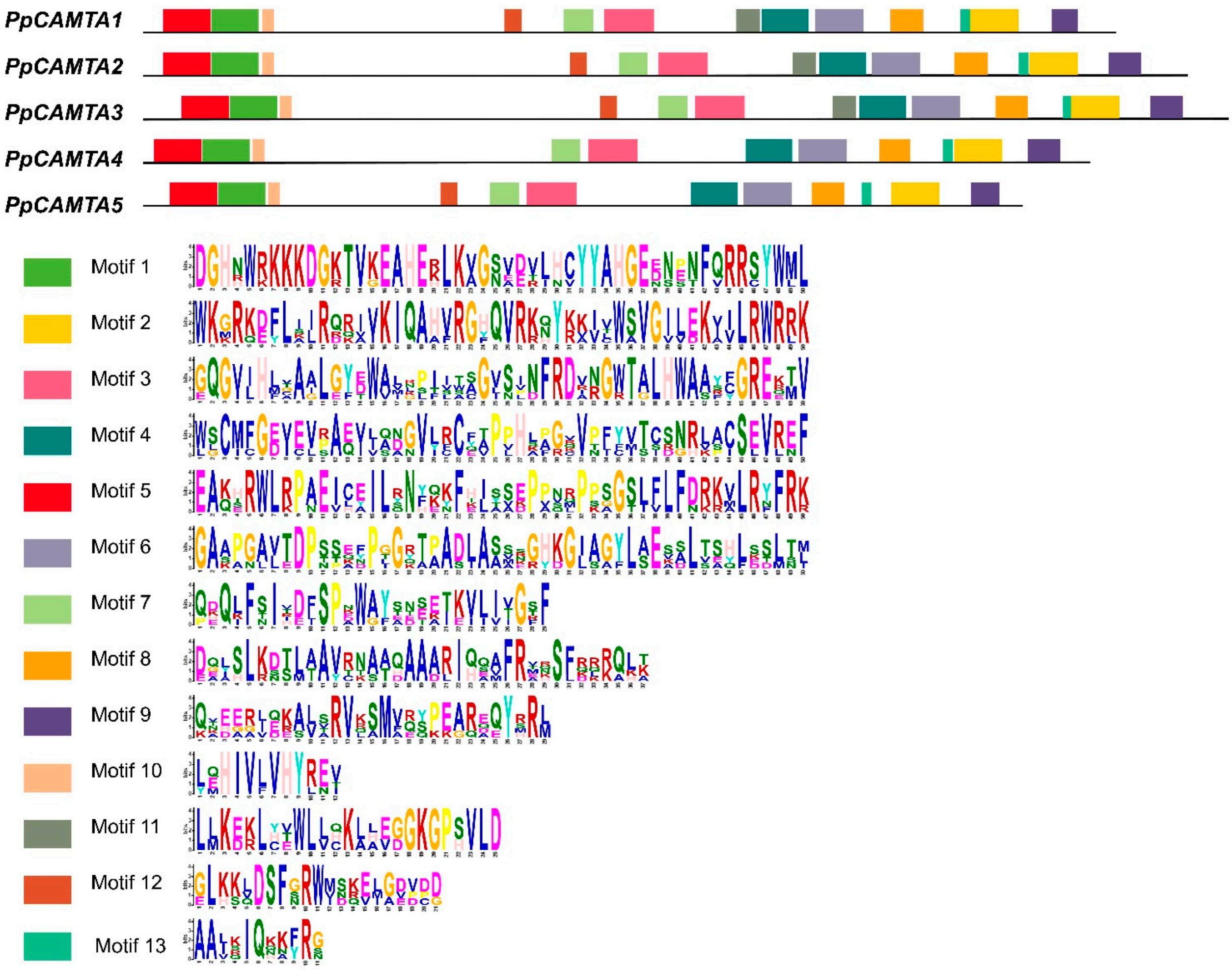

Figure S2. Transcript levels of CAMTA genes in response to UV-B and MeJA. (A) UV-B treatment. (B) MeJA treatment. Error bars represent SE (n=3). * *P*<0.05), ** *P*<0.01, *** *P*<0.001.

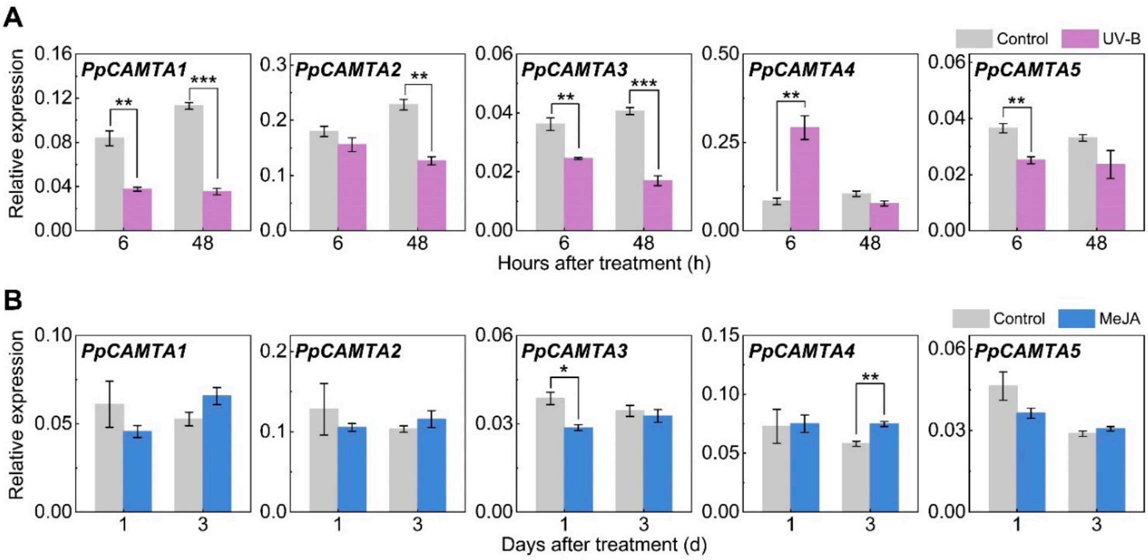

Figure S3. Expression levels of *PpCAMTA1*, *PpCAMTA2* and *PpCAMTA3* in OE-*PpCAMTAs* transgenic lines. Error bars indicate SE calculated from three biological replicates. N.D., not detected.

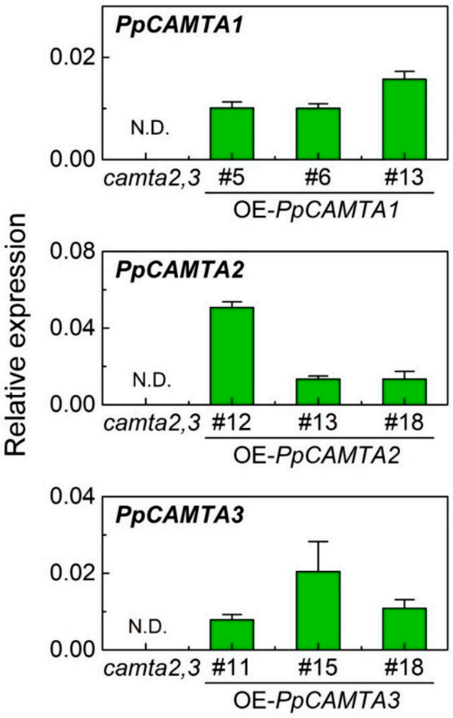

Supplement: Supplementary file 1 [file ijms-23-10500-s001.zip › ijms-1789349-SI.pdf]
